# Electrospun, Resorbable, Drug-Eluting, Nanofibrous Membranes Promote Healing of Allograft Tendons

**DOI:** 10.3390/membranes12050529

**Published:** 2022-05-18

**Authors:** Chun-Jui Weng, Yu-Chen Wu, Ming-Yi Hsu, Fu-Pang Chang, Shih-Jung Liu

**Affiliations:** 1 Department of Orthopedic Surgery, Bone and Joint Research Center, Chang Gung Memorial Hospital-Linkou, Taoyuan City 33305, Taiwan; jim_weng@hotmail.com; 2 Department of Mechanical Engineering, Chang Gung University, Taoyuan City 33302, Taiwan; wusz.master@gmail.com (Y.-C.W.); m7259@cgmh.org.tw (M.-Y.H.); 3 Department of Orthopaedics, Chang Gung Memorial Hospital, Taoyuan City 33305, Taiwan; 4 Department of Radiology, Chang Gung Memorial Hospital, Taoyuan City 33305, Taiwan; 5 Department of Pathology and Laboratory Medicine, Taipei Veterans General Hospital, Taipei 11217, Taiwan; fpchang@vghtpe.gov.tw

**Keywords:** electrospinning, drug-eluting, nanofibrous membrane, allograft, tendon repair

## Abstract

In spite of advances in medical technology, the repair of Achilles tendon ruptures remains challenging. Reconstruction with an autograft tendon provides the advantage of a higher healing rate; nevertheless, the development of donor-site morbidity cannot be ignored. We developed biodegradable, drug-eluting, nanofibrous membranes employing an electrospinning technique and evaluated their effectiveness on the healing of allograft tendons. Poly-D-L-lactide-glycolide was used as the polymeric material for the nanofibers, while doxycycline was selected as the drug for delivery. The in vitro and in vivo drug-release profiles were investigated. The biomechanical properties of allografted Achilles tendons repaired using the nanofibrous membranes were tested in euthanized rabbits at 2-, 4-, and 6-week time intervals. Histological examination was performed for the evaluation of tissue reaction and tendon healing. The level of postoperative animal activity was also monitored using an animal behavior cage. The experimental results showed that the degradable nanofibers used as a vehicle could provide sustained release of doxycycline for 42 days after surgery with very low systemic drug concentration. Allograft Achilles tendon reconstruction assisted by drug-loaded nanofibers was associated with better biomechanical properties at 6 weeks post-surgery. In addition, the animals exhibited a better level of activity after surgery. The use of drug-eluting, nanofibrous membranes could enhance healing in Achilles tendon allograft reconstruction surgery.

## 1. Introduction

The Achilles tendon is the strongest and largest tendon in the human body. Achilles tendon rupture is a common injury of the foot and ankle, which has a large influence on an individual’s level of sports activity [1]. The rate of misdiagnosis or delayed diagnosis can be as high as 25% [2,3]. Chronic injury may occur 4–6 weeks after initial injury [4,5]. Surgical treatment for primary repair is feasible in acute injury, but not in chronic injury due to the retraction of the injured tendon and superimposed fibrous tissue. Thus, chronic injury is linked to a poor prognosis. Numerous different treatment options have been used in the therapy of neglected Achilles tendon ruptures with large defects, including V-Y tendon plasty and tendon transfer or reconstruction [6]. Tendon grafts used for reconstruction include autograft, allograft, and synthetic material. Reconstruction with an autograft tendon [7] offers the advantage of a higher healing rate. Commonly employed autografts involve patellar [8], hamstring [9], and quadriceps [10] tendons. However, the development of donor-site morbidity cannot be ignored. Tendon reconstruction with allograft is free from the aforementioned disadvantage. Nevertheless, concerns regarding the healing between the allograft and the ruptured Achilles tendon exist due to the low vascularity and high weight-bearing force present during the recovery process [11,12]. 

Following tendon injury, matrix metalloproteinases (MMPs) play an important role in the healing process [13,14]. MMPs participate in the degradation and remodeling of the extracellular matrix of connective tissue [14,15,16,17]. The increase in MMP activity after tendon rupture further reduces the quality of collagen fibers [18,19,20]. Tetracycline, an established antibiotic, can inhibit the action of MMPs, thus reducing excessive degradation and remodeling of the extracellular matrix [21,22,23]. Several studies have shown reduced tendon degeneration after local and systemic use of tetracyclines [18,24,25,26]. Doxycycline is the most potent MMP inhibitor in the tetracycline family, with the ability to enhance tendon healing in the rotator cuff and Achilles tendon [27,28,29,30,31,32]. Systemic and local administrations of doxycycline have been shown to promote Achilles tendon healing after primary repair in rats [27,30,31]. Poly-D-L-lactide-glycolide (PLGA) [33,34] is an FDA-approved resorbable polymer possessing a wide range of degradation times (1–3 months) and tunable mechanical properties. The material exhibits excellent biocompatibility and has been extensively researched as a delivery vehicle for drugs, proteins, and various other biomolecules including DNA, RNA, and peptides [35,36,37,38]. Electrospinning [39], on the other hand, is an easy and versatile process that adopts electric force to stretch the charged threads of polymer solutions into fibers with a diameter in the range of micrometers to nanometers [40].

Various efforts have been completed to develop antibacterial, nanofibrous, skin scaffolds for wound-healing applications [41,42,43]. The experimental data demonstrated that electrospun nanofibers improve and accelerate the process of healing and thus offer a promising solution to the management of wounds. Additionally, electrospun nanofiber mats possess distinct, extraordinary characteristics, including excessive surface areas, a light weight, a porous network structure, and good stiffness and tensile strength, as compared to traditional fibers [44,45]. The electrospinning process can also produce nanofibrous mats with various structural assemblies, including nonwoven mesh, aligned mesh, patterned mesh, etc. [44,46,47,48]. In our previous study, we developed doxycycline-incorporated nanofibers and investigated their influence on the repair of ruptured tendons [49]. The results showed that the nanofibers promote the healing of tendon ruptures.

This study further explored whether the resorbable, doxycycline-eluting nanofibers could also enhance the healing of Achilles tendons reconstructed with allografts (grafts harvested from other individuals of the same species). Doxycycline-incorporated nanofibers were prepared using a lab-made electrospinning device. After electrospinning, the in vitro characteristics of the doxycycline-incorporated, PLGA nanofibrous mats were evaluated, and the in vivo efficacy of nanofibers was assessed using a rabbit model. Histological assay was also completed.

## 2. Materials and Methods

### 2.1. Materials

PLGA (lactide:glycolide = 50:50, Mw = 24,000–38,000 Da), doxycycline hyclate (No. D9891, Mw = 512.94 Da) and hexafluoroisopropanol were purchased from Sigma–Aldrich (St. Louis, MO, USA) [39].

### 2.2. Fabrication of Doxycycline-Loaded Nanofibers

Electrospinning experiments were completed on a lab-scale device, which involved a syringe and a needle (with an internal diameter of 0.42 mm), a ground electrode, an aluminum sheet, and a high-voltage supply [49]. PLGA (300 mg) and doxycycline (75 mg), at concentrations of 30% (*w/v*) and 7.5% (*w/v*), respectively, were initially blended with as-purchased hexafluoroisopropanol (1 mL) using a magnetic stirrer for 2 h. The PLGA/drug solution was delivered and electrospun by a syringe pump with a volumetric flow rate of 0.1 mL/h. The travel distance between the tip of the needle and the ground electrode was 15 cm, and the voltage applied to the polymer solutions was +15 kV. After electrospinning, the PLGA/doxycycline nanofibers were gathered on the aluminum sheet. All nanofibrous samples were prepared at ambient temperature (25 °C) and 65% relative humidity. Nanofibrous membranes with a thickness of approximately 0.2 mm (measured using a Mitutoyo micrometer, Tokyo, Japan) were thus obtained; each membrane took approximately 3 h to be successfully electrospun. All electrospun nanofibers were placed in a vacuum oven at 40 °C for 3 days to vaporize the residual solvents.

We used a field-emission scanning electron microscope (SEM; JSM–7500F; Joel, Tokyo, Japan) to evaluate the doxycycline-embedded fibers after they were coated with gold. A total of 50 arbitrarily selected nanofibers were characterized employing commercial Image J image code (National Institutes of Health, Bethesda, MD, USA) for each sample to determine the diameter distribution (*n* = 3).

The thermal properties of the pristine PLGA, doxycycline, and doxycycline-loaded PLGA nanofibers were investigated using a TA-DSC25 differential scanning calorimeter (DSC) (TA Instruments, New Castle, DE, USA). Nitrogen was used as the gas of choice in the analysis, and the built-in software was used to process the data. The scanning temperature ranged from 30–350 °C, while the nanofibrous specimens were heated at 10 °C/min.

### 2.3. In Vitro Release Patterns of Pharmaceuticals

The release pattern of the doxycycline from the drug-loaded, biodegradable, nanofibrous membranes was obtained through an in vitro elution method. Samples cut from the electrospun mats (1 cm × 1 cm) were placed inside glass tubes (1 specimen per tube; 3 specimens were employed for each test trial, *n* = 3) along with 1 mL of phosphate buffer solution in each tube. After the glass tubes were incubated for 24 h at 37 °C, the eluent was collected and analyzed. During the next 24 h, 1 mL of fresh phosphate-buffered saline was added, and the procedure was repeated for 42 days. The levels of doxycycline in the mixtures were evaluated through high-performance liquid chromatography (L-2200R multi-solvent delivery system; Hitachi, Tokyo, Japan).

### 2.4. Animal Experiments

A total of 24 white New Zealand rabbits (weight: 2500–3000 g) were included in the study; 3 animals were used for in vivo drug-release testing, while the remaining 21 animals were employed for the tendon biomechanical and postoperative activity experiments. A total of 3 of the 21 animals did not undergo surgery and served as the healthy group in the activity test. Table 1 lists the number of animals in different tests. The entire animal-related process acquired approval from the Institutional Animal Care and Use Committee of Chang Gung University (IACUC Approval No.: CGU107-168, Approval date: 28 November 2019), and all enrolled rabbits were cared for according to the regulations of the Department of Health and Welfare, Taiwan.

For anesthesia, the rabbits were placed into a transparent chamber (40 cm × 20 cm × 28 cm) filled with vaporized isoflurane (Aesica-Queenborough, Queenborough, Kent, UK) with a concentration of 2% (*v*/*v*). After anesthesia, each rabbit was placed onto a disinfected, disposable sheet with the continuous administration of vaporized isoflurane through a mask.

**Figure 1:**
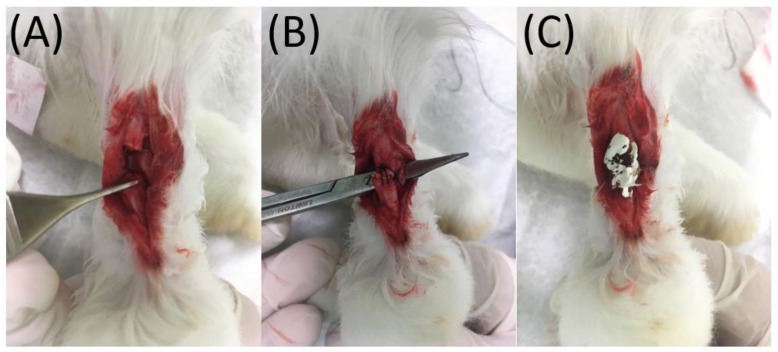
(**A**) The Achilles tendon was cut using a blade. (**B**) The tendon was primarily repaired with Dexon sutures, and (**C**) enveloped with doxycycline-embedded nanofibers.

After disinfecting the right leg of each rabbit with 75% alcohol, local anesthesia was induced through a local injection of 2% xylocaine (1 mL). A 4 cm longitudinal skin incision was made with a No. 10 blade over the posterolateral side of the right leg. Gentle dissection of the Achilles tendon was performed circumferentially with Mosquito (Figure 1). In case of active bleeding, direct pressure with a disinfected gauze pad was applied to the wound for hemostasis. The Achilles tendon (0.5 cm) was harvested from the rabbit through a horizontal cut proximally and distally over the middle portion of the tendon for further transplantation to another rabbit. Subsequently, a tendon (0.5 cm) from another rabbit was transplanted to this harvest site, end-to-end repaired proximally and distally using 3-0 Dexon (Johnson & Johnson, New Brunswick, NJ, USA) sutures. Before implantation, the biodegradable, nanofibrous membranes were disinfected by ethanol at a concentration of 75%. No obvious influence of disinfection on the membranes was observed. In the experimental group, a biodegradable, nanofibrous membrane (30 mm × 30 mm × 0.2 mm, L × W × T) weighing approximately 12 mg was wrapped around the transplanted/repaired tendon circumferentially and fixed with 5-0 Dexon (Johnson & Johnson, New Brunswick, NJ, USA) sutures at each end of the membrane to make sure that the membrane would not be dislodged. Next, the superficial skin was repaired with 3-0 Nylon sutures. In the control group, the surgical wound was repaired directly with 3-0 Nylon sutures without any membrane. In all rabbits, neomycin ointment was applied to the sutured wounds to prevent infection. After the operations, all animals were permitted to migrate freely in their individual cages and given standard rabbit chow and sterilized drinking water ad libitum. The temperature was kept at 25 °C, and the humidity was maintained at 65%.

### 2.5. In Vivo Drug Release

Three rabbits were enrolled for the in vivo drug-release experiments. At 1, 3, 14, 28, and 42 days post-implantation, the rabbits were euthanized, and the tissues around the membrane were sampled (*n* = 3). Meanwhile, blood sampling (1 mL) was also performed by syringe via puncture on the marginal ear vein (*n* = 3). The blood sample was freshly centrifuged at 2500 rpm for 15 min so as to separate plasma from whole blood. The drug levels of doxycycline in the tissue and plasma were evaluated using Hitachi L-2200R high-performance liquid chromatography (Tokyo, Japan) [50,51]. The mobile phase contained acetonitrile, distilled water, and phosphoric acid (pH = 2.5) (50/20/30, *v*/*v*/*v*). The absorbency was monitored at 245 nm, and the flow rate was 1.0 mL/min.

### 2.6. Post-Operation Activity Testing

**Figure 2:**
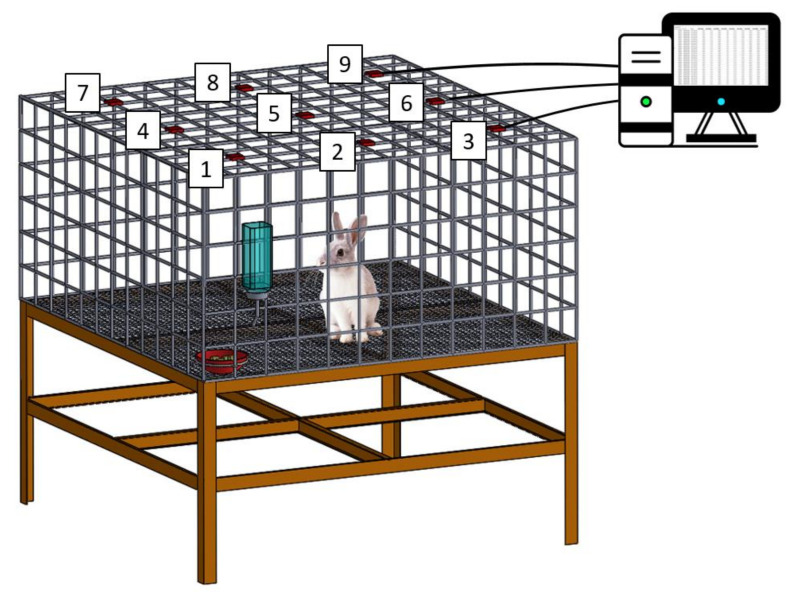
Schematic of the animal behavior cage. The cage (120 cm × 120 cm × 60 cm) was divided into nine symmetrical square areas. One diffusion-scan type photoelectric switch sensor was placed above each square area.

Nine rabbits were used in the activity test, including three normal rabbits, three in the experimental group, and three in the control group (surgery only, no membrane). Following the operation, each rabbit was raised in an animal behavior cage (Figure 2; manufactured in our laboratory) for 1 week to record the level of postoperative activity. The cage (120 cm × 120 cm × 60 cm) was divided into nine symmetrical, square areas. one diffusion-scan type photoelectric switch sensors were placed above each square area (HP100-A1; Azbil Corp., Tokyo, Japan). When the rabbit moved from one square area to another, the corresponding “arriving” sensor would be triggered. The number of times the sensor was triggered was monitored and recorded for 1 week. Thereafter, the rabbit was placed in its original cage for further animal care. The activities of the experimental, control, and healthy groups of animals were assessed (3 rabbits in each group, *n* = 3); control rabbits that underwent surgery only (no membrane), experimental rabbits that underwent surgery and the implantation of the doxycycline-loaded nanofibers, and healthy rabbits that did not undergo surgery).

### 2.7. Biomechanical Strength Testing

A total of 18 animals were enrolled in the tensile strength study. To minimize the use of animals, 6 rabbits from the activity test, after being monitored for 1 week, were re-included in this analysis. The rabbits were euthanized by intravenous injections of lidocaine (10 mL) 2, 4, and 6 weeks after surgery. The right Achilles tendons and the left healthy tendons (with an approximate length of 5 cm) were harvested for further biomechanical strength testing (*n* = 3). The tensile test was performed using a Lloyd tensiometer (AMETEK, Largo, FL, USA) equipped with a 2.5 kN load cell, according to the ASTM D638 standard. The tendon was clamped at both ends onto the tensiometer and pulled at a speed of 6 mm/min for 10 cm.

### 2.8. Histological Analysis

Retrieved tendons were embedded in 10% formalin and fixed with paraffin. Next, the retrieved tendon was sectioned in the frontal direction (thickness: 4 μm). Hematoxylin and eosin staining was used for histological evaluation. We evaluated six different parameters, including fiber structure, fiber arrangement, nuclei rounding, white blood cell accumulation, vascularity, and cell density. Each parameter was scored from 0 to 3; with 0 representing normal and 3 representing totally abnormal. With this semi-quantitative scoring system [52], histologic analysis was performed by an independent pathologist blinded to the grouping.

### 2.9. Statistical Analysis

We used the paired *t*-test for statistical evaluation between different two-group combinations. Statistical significance was set at *p*-values below 0.05.

## 3. Results

### 3.1. Assessment of Spun Doxycycline-Loaded Nanofibers

**Figure 3:**
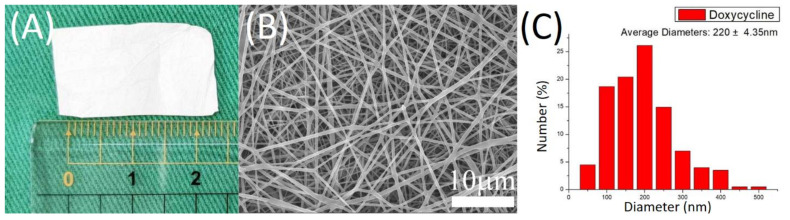
(**A**) Gross view; (**B**) scanning electron microscope (SEM) image of doxycycline-loaded poly-D-L-lactide-glycolide (PLGA) nanofibrous membranes (magnification: ×5000); (**C**) size distribution of nanofibers.

**Figure 4:**
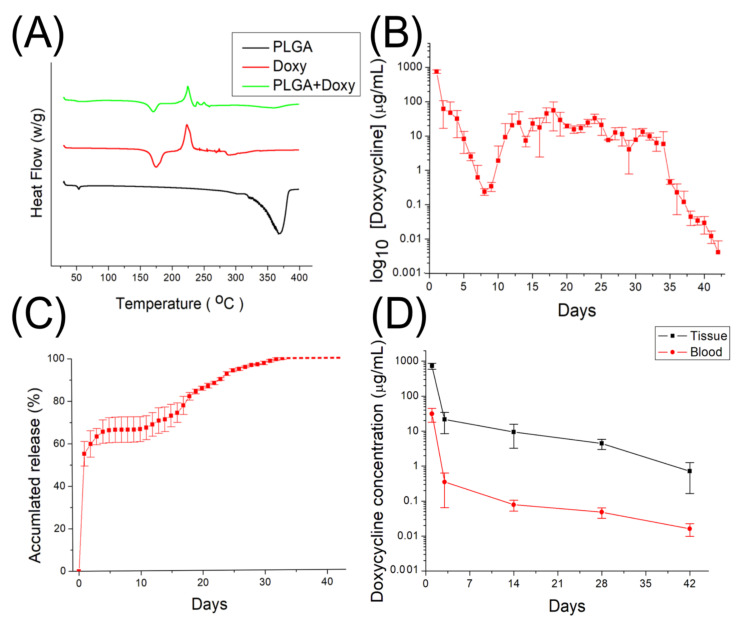
(**A**) Differential scanning calorimetry (DSC) assay of pure PLGA, doxycycline, and doxycycline-embedded, PLGA, nanofibrous membranes; (**B**) daily release, and (**C**) cumulative discharge of doxycycline in vitro from the nanofibers; (**D**) in vivo release of doxycycline in tissues and bloods over time.

Figure 3A shows the gross view of the electrospun nanofibers, while Figure 3B displays the SEM image of the doxycycline-embedded nanofibers, and Figure 3C illustrates the size distribution of the nanofibers. The calculated diameter distribution of spun nanofibers was 200 ± 4.3 nm. The thermal properties of isolated PLGA, doxycycline, and doxycycline-loaded PLGA nanofibers were assessed, and the results are displayed in Figure 4A. Due to the inclusion of doxycycline, the thermogram of the drug-loaded polymeric fibers was similar to the drug instead of the polymer. The endothermal peak of doxycycline at 369 °C disappeared after being incorporated into the PLGA matrix [53,54], demonstrating the successful embedding of the doxycycline into the PLGA nanofibrous mats.

### 3.2. Doxycycline Release

Figure 4B,C display the daily and cumulative discharge profiles of doxycycline from the nanofibrous membranes. The profile showed a triphasic release: a burst release at day 1, a second major peak of discharge at day 17, a few minor peaks at 13 and 24 days, and a progressive reduction in drug elution thereafter. Moreover, the drug-loaded, nanofibrous membranes provided sustained in vitro release of doxycycline for over 42 days.

Figure 4D displays the elution profiles of in vivo doxycycline in blood and tissue. The local concentration of the drug at the tendon was high for 6 weeks, whereas the systemic drug concentration was markedly lower. Different from that in vitro, the in vivo profile showed a bi-phasic discharge, namely an initial burst and then a gradual diminishing release.

### 3.3. Biomechanical Strength Study

**Figure 5:**
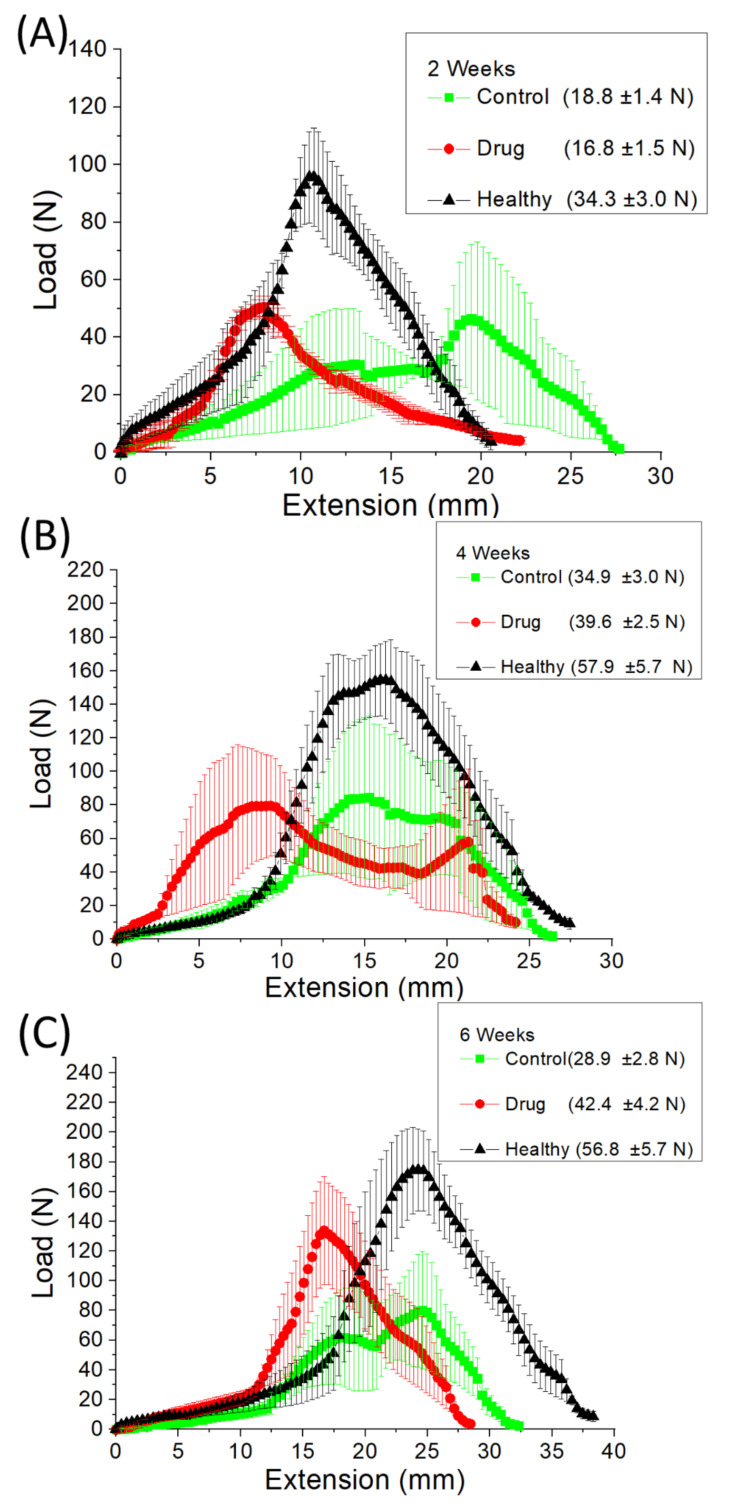
Tendon strengths at (**A**) 2, (**B**) 4, and (**C**) 6 weeks post-surgery in the healthy, doxycycline-loaded, and control (surgery only, no membrane) groups.

At 2 weeks (Figure 5A), the maximum loads of the control (surgery only, no membrane) group and the doxycycline-loaded group were similar and significantly lower than that of the healthy group (*p* < 0.05). Similar results were noted at 4 weeks (Figure 5B). The maximum loads of the doxycycline-loaded and control groups were lower than that of the healthy group (*p* > 0.05). At 6 weeks after surgery, the maximum load was markedly lower in the control group (Figure 5C). The maximum loads of the doxycycline-loaded and healthy groups were comparable (*p* > 0.05), testifying to the extraordinary ability of doxycycline-loaded nanofibers in promoting the healing of the reconstructed, allografted Achilles tendons.

### 3.4. Animal Activity

**Figure 6:**
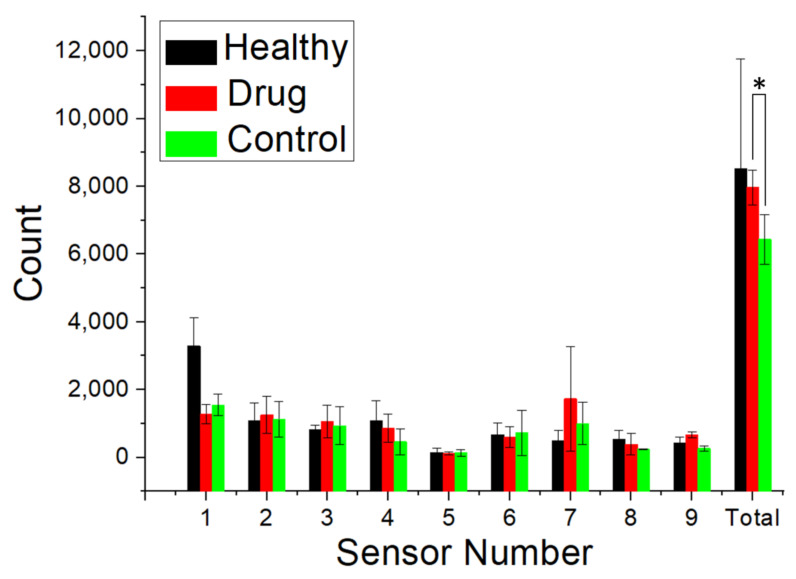
Activity counts of the rabbits in the healthy, doxycycline-loaded, and control (surgery only, no membrane) groups (* *p* < 0.05). The photoelectric switch sensors were triggered 8519 ± 3235, 7964 ± 510, and 6437 ± 732 times in the healthy, doxycycline-loaded, and control (surgery only, no membrane) groups, respectively. Rabbits undergoing allograft Achilles tendon reconstruction with a doxycycline-loaded, biodegradable, nanofibrous membrane had similar levels of activity to healthy rabbits and higher activity levels compared to control rabbits.

The activity of the animals post-operation was evaluated using an animal behavior cage. The total number of times the sensors were triggered in each group is shown in Figure 6, with higher numbers representing better animal activity. The difference between the doxycycline-loaded and control groups was statistically significant (*p* < 0.05). Nonetheless, there was no significant difference between the doxycycline and healthy groups. Therefore, rabbits undergoing allografted Achilles tendon reconstruction with a doxycycline-loaded biodegradable nanofibrous membrane had a level of activity similar to that of the healthy rabbits and higher activity levels compared to those of the control rabbits.

### 3.5. Histological Study

**Figure 7:**
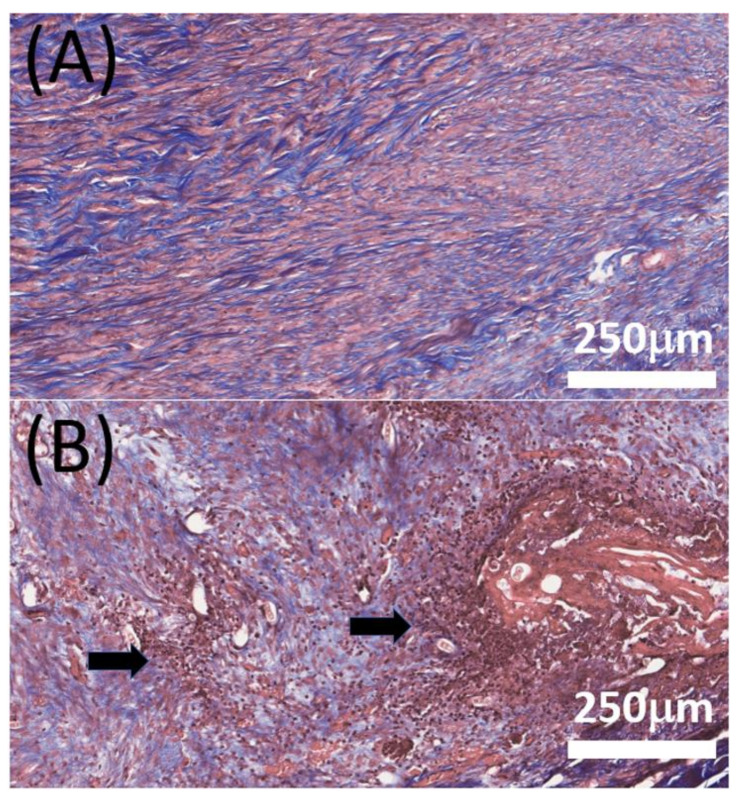
H&E staining of the Achilles tendon at 4 weeks post-surgery. (**A**) Doxycycline group; (**B**) Control (surgery only, no membrane) group. (Magnification: ×160, Pink represents collagen fiber. Arrow: white blood cell accumulation, which indicates inflammation.). Abbreviation: H&E, hematoxylin and eosin.

Figure 7 shows images obtained after hematoxylin and eosin staining. There were no significant differences noted in terms of tendon cell maturation, proliferation, or arrangement between the doxycycline-loaded and control (surgery only, no membrane) groups at any timepoint. However, at 4 weeks post-operation, the images revealed severe inflammation with the extensive accumulation of white blood cells in the control group and only mild inflammation in the doxycycline-loaded group. In addition, the collagen-fiber orientation was scored with a semi-quantitative grading system [52]. However, no significant difference was found in collagen fiber.

## 4. Discussion

Allograft tendon reconstruction has become increasingly important, especially when there is a shortage of suitable available local tissue. The advantages of using allograft tissue involve the avoidance of donor site morbidity, high tensile strength, reduced operational time, smaller surgical incisions, and a low risk of arthrofibrosis [55,56]. However, the healing between the allograft and ruptured tendon remains a major concern for surgeons. The role of MMPs in the healing process has been widely studied [13,14,57]. Increased MMP levels lead to poor collagen quality during tendon healing [20,58,59]. Doxycycline, as the most widely studied drug among the MMP family, enhances healing in the primary repair of Achilles tendons. Currently, there is no gold standard in terms of the route of administration (systemic or local), duration of treatment, or concentration of doxycycline.

In the literature, oral administration of doxycycline has been found to maintain an effective drug concentration over a long period of time [24,27]. Kessler et al. [27] proposed that administration of doxycycline through oral gavage for 4 weeks significantly inhibited MMP activity by 60% in rats after Achilles tendon transection and repair. Improved collagen fibril alignment and better biomechanical properties were noted in the extended doxycycline treatment group. In their study, the serum level of doxycycline was 6 µg/mL. Nguyen et al. [30] used oral doxycycline (10 mg/kg daily) for the treatment of surgically repaired and unrepaired Achilles tendons in rats. They found that doxycycline improved the biomechanical properties of Achilles tendons at 3–6 weeks after surgical repair. Nevertheless, the serum concentration of doxycycline was not evaluated in their study. Pasternak et al. [60] used a rat model with Achilles tendons transected and left unrepaired. Oral doxycycline was administered at a mean serum concentration of 3.4 µg/mL. Decreased force at failure was noted at 5, 8, and 14 days post-surgery in the doxycycline group. However, there was no significant difference noted in stiffness or stress at failure at all three time points. Despite its effectiveness, various adverse effects (e.g., gastritis, esophageal erosion, heartburn, nausea, or vomiting) have been reported [51] after long-term systemic use of doxycycline.

The progress of nanotechnology in drug-delivery provides the potential for enhanced treatments with targeted delivery and minimized side effects through the employment of designed nanomaterials, as well as via the formation of delivery systems from nanoscale molecules and/or vehicles. In this study, degradable nanofibers were manufactured for the sustainable discharge of doxycycline into the target area for allograft tendon reconstruction. Owing to their excessive surface-area-to-volume ratio, nanofibers offer an advantageous vehicle for the transport of water-insoluble or poorly soluble pharmaceuticals. Another great advantage of nanofibrous mats lies in their similarity with the natural fibrillary extracellular matrix (ECM), which facilitates cell attachment and proliferation for biomedical applications.

In this work, we prepared degradable, PLGA nanofibers that provide the sustained release of doxycycline. The experimental results showed that electrospun nanofibers exhibit a tri-phasic drug-release behavior (Figure 5) in vitro. The discharge of drugs from a resorbable polymeric vehicle generally consists of three various phases: a burst, a diffusion-dominated phase, and a degradation-governed phase. In the electrospinning procedure, most drugs are dispersed in the bulk of the polymeric frame. However, certain drugs may be allocated on the surface of nanofibrous membranes, resulting in an initial burst. After the burst, the discharge of pharmaceuticals is governed and affected by diffusion. A major secondary peak elution at 17 days and few minor peaks at 13 and 24 days were observed, after which the drug level gradually decreased. Meanwhile, the restorable nanofibers displayed a bi-phasic release in vivo: an initial burst and a gradual diminishing release. This might be due to the fact that the in vivo metabolism is slower than the in vitro elution. A second peak release was thus not noted. Overall, the results indicated that electrospun, polymeric, drug-loaded membranes can discharge high concentrations of doxycycline for over 42 days, both in vitro and in vivo, while the drug levels remained much lower in blood. Additionally, the in vivo animal tests also demonstrated that the doxycycline-eluting nanofibers promote Achilles tendon reconstruction with allografting.

Previous studies demonstrated that the effect of doxycycline on tendons becomes significant from at least 3 weeks after surgery [24,25,27,47]. It appears that doxycycline requires a longer period of time to achieve a statistically significant influence on the healing process of allografted tendons. Pasternak et al. [29] used doxycycline-coated sutures to improve the holding capacity and found that 73% and 96% of the doxycycline was released from the surface of the sutures after 24 and 72 h, respectively. In contrast, 56% and 63% of embedded doxycycline was released, respectively, from the nanofibers in our study. By using the PLGA-based membrane, we reached a local concentration of at least 1 µg/mL doxycycline for 42 days and a systemic concentration of doxycycline under 0.1 µg/mL after 3 days. Oral administration is most common for doxycycline, and an oral dose of 100 to 200 mg is absorbed rapidly. Previous data showed that doxycycline is detectable in the plasma at 15 min after administration, and a peak concentration of 1700 to 5900 µg/mL in the blood can be measured after 2 to 3 h [61]. Despite the drug concentration in the rabbit’s tissue being high (approximately 1000 µg/mL, Figure 4D) at day 1, it was still inferior to that orally administered. In particular, the drug level in the animal’s blood (30 µg/mL) was much lower than that caused by oral administration, demonstrating the advantage of local drug delivery through nanofibers. Additionally, the experimental results in Figure 7 proved that doxycycline-embedded, biodegradable, nanofibrous membrane is effective at enhancing tendon strength 6 weeks after Achilles tendon reconstruction with allografting in rabbits. There were no drug-related or major complications observed in this analysis. Besides, less white blood cell (WBC) accumulation was noted through histologic evaluation, which might be attributable to the anti-inflammatory effect of doxycycline [62,63]. These findings indicate that the biodegradable, nanofibrous membrane can maximize the positive effect of doxycycline on allograft tendon healing, while minimizing the side effects associated with the long-term usage of doxycycline. Nevertheless, the in vivo results in Figure 7 suggest that, compared to the healthy tendons, the repaired, allografted tendons exhibited the maximum strength at lower extensions, namely, the peak strengths shift to the left on the load-extension curves. This illustrates that the reconstructed allograft tendons possessed inferior extensibility in comparison with the healthy tendons. Additionally, the toughness, the area below the load-extension curves, of the reconstructed allograft tendons was also found to be smaller than that of the healthy tendons.

There are limitations associated with this study. First, the number of animals employed in this work has been low. Second, we did not examine the relationship between drug concentration and tendon healing so as to optimize the drug loadings in the nanofibers for tendon reconstructions. Third, the thermo-interaction between PLGA and doxycycline remained unclear. Fourth, more research will be needed to better comprehend the microscopic mechanism leading to the reduction of extensibility in reconstructed allografts. Finally, the relevance of the present findings to tendon reconstruction with allograft in humans remains uncertain. These will be the topics of our future research.

## 5. Conclusions

We evaluated the efficacy of doxycycline-loaded, biodegradable nanofibers for allograft tendon reconstruction in rabbits. The experimental results showed that the nanofiber can provide persistent release of doxycycline for 42 days. The concentration of doxycycline was markedly higher in local tendon tissue than in blood, indicating a very low systemic drug effect. Despite the doxycycline concentration in the rabbit’s tissue being high at day 1, it was still inferior to that when orally administered. In particular, the drug level in the animal’s blood was much lower than that found when the drug is administered orally, demonstrating the advantage of local drug delivery through nanofibers. In addition, animals receiving allograft tendon reconstruction with doxycycline-loaded nanofibers exhibited stronger tendons and higher levels of activity after surgery. The reconstructed allograft tendons showed less extensibility than the healthy tendons. Histological assays did not display any notable tissue inflammation. Resorbable nanofibers with sustained discharge of doxycycline demonstrated the potential for the reconstruction of Achilles tendons via allografting.

## Figures and Tables

**Table 1:** Number of animals enrolled in the in vivo tests.

Test	No. of Animals Employed	Annotation
Drug concentration	3 **Subtotal: 3**	The drug levels at 1, 3, 14, 28, and 42 days post-implantation in the surrounding tissues and blood were evaluated (*n* = 3).
Activity	Health ×3 Control ×3 Experimental ×3 **Subtotal: 3**	The animals used in the control and experimental groups (6 rabbits) were re-included in the biomechanical tests.
Biomechanical	Health ×9 (right legs of the animals) Control ×9 (3 from the activity test) Experimental ×9 (3 from the activity test) **Subtotal: 18**	A total of 6 rabbits from the activity test, after being monitored for 1 week, were re-included in this analysis. The rabbits were euthanized at 2, 4, and 6 weeks after surgery (*n* = 3). The right Achilles tendons and the left healthy tendons were harvested for further biomechanical strength testing.
Total	24	

## Data Availability

Data is contained within the article.
